# U. S. Experiment Station Veterinarians

**Published:** 1897-10

**Authors:** 


Secretary.

### U. S. EXPERIMENT STATION VETERINARIANS.

The United States Experiment Station Veterinary Medical Association met at Nashville, Tenn., Tuesday evening, September 7th, and organized by electing the following officers: Dr. C. A. Cary, President, and Dr. A. T. Peters, Secretary. Dr. James Law, of New York; Dr. S. B. Nelson, of Washington, and Dr. J. W. Connoway, of Missouri, were appointed as an Executive Committee, of which the officers of the Association are ex-officio members.

The objects of the Association are to enable experiment station men to get together in their States and make experiments. A constitution and by-laws were adopted, and, under a suggestion of the Executive Committee of the United States Veterinary Medical Association, it was ordered that the proceedings of this Association be printed with the proceedings of the United States Veterinary Medical Association.

